# The Potential for Topical Probiotic Treatment of Chronic Rhinosinusitis, a Personal Perspective

**DOI:** 10.3389/fcimb.2017.00530

**Published:** 2018-01-12

**Authors:** Anders U. Cervin

**Affiliations:** ^1^Faculty of Medicine, The Garnett Passe and Rodney Williams Memorial Foundation Chair in Otolaryngology, (Rhinology), University of Queensland, Brisbane, QLD, Australia; ^2^Department of Otolaryngology, University of Queensland Centre for Clinical Research, Brisbane, QLD, Australia; ^3^Department of Otolaryngology, Head and Neck Surgery, Royal Brisbane and Women's Hospital, Herston, QLD, Australia; ^4^Royal Brisbane and Women's Hospital Clinical Unit, Herston, QLD, Australia

**Keywords:** probiotic, chronic sinusitis, clinical trial, microbiota, microbial dysbiosis, airway

## Abstract

This review describes the rationale for topical probiotic intervention, the obstacles we are facing and a strategy for future research in the use of probiotics to modify CRS symptoms and disease expression. Recent advances in molecular microbiology has revealed a plethora of microbial DNA in the nasal cavity and sinuses of healthy subjects as well as in chronic sinusitis (CRS) patients. An infection is today rather seen as an imbalance between the commensal microbiome and the bacterial pathogens, resulting in a reduction in commensal bacterial diversity, combined with an increase in the growth of microbes eliciting an inflammatory response. This will in turn lead to the clinical symptoms of sinusitis. Probiotics (microorganisms that confer a health benefit) can be used either as a form of living antibiotics treatment, or as an immune-modulatory intervention. Topical probiotics, which is the focus of this review, have shown efficacy in a limited number of trials in otitis media and tonsillitis, but to date not in CRS. Although bacterial interference capacity against pathogens can be determined in *in vitro* experiments, it may not translate to a health benefit. This limits the role of laboratory research in identifying probiotic strains with a clinical benefit. To gain more clinical experience without further delay, I recommend future research to focus on empirical clinical trials in well-defined CRS patient populations and study the underlying mechanisms in more detail once a clinical benefit has been established.

## The current concept of chronic rhinosinusitis and limitations in relation to researching the microbiome

Chronic rhinosinusitis (CRS) is responsible for significant morbidity and health care costs across the globe. In spite of, the advancement of surgical methods and medical treatment little improvement has been seen in in the last 2 decades and it is estimated that 30% of CRS sufferers have symptoms not controlled by guideline therapy (Fokkens et al., 2012). Furthermore, I would argue that the present classifications system, based on ocular observation only, CRS with or without nasal polyps, is outdated and provides a very restricted view of the heterogeneous pathophysiology responsible for CRS symptoms. It is time for a fundamental re-design of the current guidelines with emphasis on traits that can be measured and treated, such as the type of inflammation present in the sinuses. Stratification according to the degree of eosinophilia and or neutrophilia in the tissue would provide a better guide for the clinician.

## Which CRS are we aiming to treat with probiotics?

It is in this context that we should consider the prospect of using probiotics (microorganisms that confer a health benefit) in the treatment of CRS. The first question is therefore; which CRS are we aiming to treat with probiotics? To date, the primary concept of topical probiotic trials has been to outcompete pathogens on the mucosal surface. As such the subgroup of CRS patient we are likely to target would be the one with well-defined pathogens colonizing the nasal cavity and the sinuses. Although, the potential for probiotic strains to have effects on the regulation of immunity as well as wound healing should not be overlooked (Mohammedsaeed et al., 2015). If the latter effects are conveyed by the same strains effective in outcompeting pathogens is not known. In this context however, I will focus on the potential of probiotic intervention to interfere with disease causing microorganisms.

## The role of the commensal microbiome in the upper airway

### The commensal microbiome and the immune system

I will briefly mention the role the commensal microbiome has in developing our immune system to a functioning defense system distinguishing self from non-self without causing undue tissue harm to the host. It seems that proper exposure to microbiota early in life is paramount to hone the immune system as exemplified in a study using the Danish birth register where 2 million term children delivered either by natural birth or cesarean was compared for inflammatory disorders later in life, and it is evident that children born by cesarean delivery had significantly increased risk of asthma, systemic connective disorders and inflammatory bowel disease (Sevelsted et al., 2015). These data indicate that the first microbial exposure, when passing through the birth canal is paramount in developing the immune system. Inflammatory disorders have also been shown to be less common, in children who suck their thumb and bite their nails as well as in children whose parents clean their pacifier by sucking on it, an unintentional microbial transplant (Hesselmar et al., 2013; Lynch et al., 2016). These epidemiological studies show a link between early exposure to microorganisms and a lower incidence of inflammatory disorders later in life and it is referred to as “the hygiene hypothesis.” Findings suggests that a diverse microbiome in the gut is likely to be a key factor.

The microbiome of the airway has been less investigated, but it there have been suggestions that there is a healthy airway core microbiome (Erb-Downward et al., 2011), however most researchers still find it difficult to distinguish between healthy and diseased by analyzing the microbiome only (Psaltis and Wormald, 2017). The overall bacterial burden is similar in healthy subjects compared to CRS patients. Where a clear pathogen can be identified there seems to be a reduction in the overall bacterial diversity on the airway mucosal surface, suggesting that diversity is an indicator for a healthy microbiome (Ramakrishnan et al., 2013, 2016). There is still no consensus on what constitutes a healthy microbiome in the sinuses, although certain phyla (the major lineages of the domain bacteria) have been identified in healthy controls and among those are; Firmicutes such as different strains of *Lactobacillus*, Actinobacteria, where you will find different strains of *Propionibacterium*, and Bacteroidetes (Abreu et al., 2012; Aurora et al., 2013; Ramakrishnan et al., 2013). To complicate matters further some species may act as commensal under certain circumstances, for example in low abundance or in concert with other microbes, where under different circumstance the same species may induce an inflammatory response. This seems to be the case with *S. Aureus* where in low abundance it may act as a commensal (Schwartz et al., 2016). Our own experiments confirm that certain strains of *Propionibacterium*, may act as a commensal, but under certain circumstances enhance the growth of pathogens (unpublished data).

## Potential mechanisms by probiotics to restore sinus health

What tools do commensal microbiome have available to outcompete pathogens? *Lactobacilli*, probably the most common probiotic, used as a food preservative in fermented food, produces lactic acid and thus lowers pH. It is well known that, for example; *Pseudomonas* does not thrive in an acid environment. Other interference mechanisms include competing for cell surface receptors, thus inhibiting adherence of pathogens, producing antibacterial peptides and other antibacterial metabolites such as hydrogen peroxide. It is also likely that an abundance of commensal bacteria may starve the pathogens for nutrients (exploitative competition). For a more detailed review of this complex topic, see Stubbendieck et al. (Stubbendieck and Straight, 2016). From the gut, there is evidence that the commensal microbiome has direct action on epithelial cells, stabilizing tight junctions, reducing production of pro-inflammatory cytokines and preventing apoptosis as well as interacting with lymphocytes with an increased production of anti-inflammatory cytokines such as IL-10 and IL-12 (Rosenberg et al., 2016).

## Evidence in the literature for topical probiotic treatment of upper airway infection

Unfortunately, there is a paucity of data regarding topical probiotic treatment of CRS, with only one placebo controlled trial available. There are several clinical trials however in otitis media as well as a trial in recurrent tonsillitis where topical bacterial interference has proven successful. There is also a small case series where MRSA carriers have been successfully treated with a combination of probiotic nasal spray and mouth wash. Table 1 outlines these trials.

**Table 1:** Topical probiotic intervention in ENT related disease.

**References**	**Indication**	**Treatment**	***n***	**Duration**	**Outcome**
Roos et al., 1993	Recurrent Tonsillitis	*Alpha Streptococci* (4 strains) Mouth spray	130	10 days	Recurrence 2% in treatment group and 23% in placebo group
Roos et al., 2001	Acute and Secretory Otitis Media	*S. mitis, S. sanguinis, S. oralis*, Nasal spray	103	10 + 10 days during 3 months	Significant reduction of AOM and SOM
Tano et al., 2002	Acute Otitis Media	*S. mitis, S. sanguinis, S. oralis*, Nasal spray	43	120 days	No effect
Skovbjerg et al., 2008	Secretory Otitis Media	*S. sanguinis, L. Rhamnosus*, Nasal spray	60	10 days	Significant effect on SOM
Roos et al., 2011	MRSA Staph Aureus carriers	*L. paracasei Ssp. paracasei, L. rhamnosus, L. plantarum*, Nasal spray and Oral suspension	7	150–300 days	MRSA eradicated in 5 out of 7 patients
Marchisio et al., 2015	Acute Otitis Media	*S. Salivarius*, Nasal spray	97	For 5 days per month for 3 months	If nasopharynx successfully colonized, a significant effect on AOM
Mårtensson et al., 2017	CRS	*Honey bee microbiome* Nasal spray	20	14 days	No effect

What is evident when you study the clinical trials in detail is the lack of side effects, it seems that topical probiotics are safe, although the number of participants is still small, in total 460 patients reported in the studies above. Furthermore, except for the Martensson et al. study, the strains are all derived from humans, either oral or nasal microbiome and may already be present in the host.

The effects of a probiotic strain are specific rather than a general trait of a genus such as for example; *Lactobacilli*. This makes the whole process of identifying potential probiotic candidates much more complex and time consuming. Overall it seems that *Streptococci* strains are more successful than *Lactobacilli* strains. Theoretically, *Streptococci* strains may be more prone to possible complications such as endocarditis, which is caused by low virulence *Streptococci viridans*, especially seen in patients with valve disorders or immune deficiency. I have not yet come across of a report suggesting that probiotic treatment have been responsible for such a course. However, it is important that all probiotic candidates are screened for antibiotic resistance and virulence genes.

Duration of treatment in past trials varies from 10 days up to 300 days and it is difficult to draw any conclusions on what constitute an effective treatment period. Although it seems that a duration of around 10 days is effective in several studies. Adherence of the probiotic strain to the mucosal surfaces may play a crucial role here, if this happens effectively, a shorter treatment duration may be all that is needed. One could however also speculate that a probiotic strain capable of changing pH or producing antibacterial substances could provide efficacy without adherence, if the probiotic is replenished regularly.

The Roos et al. case series on difficult to treat MRSA, is especially interesting as all patients had been carriers for over a year. This demonstrates that probiotic can be effective in spite of the target strain being resistant to multiple antibiotics. It shows promise for the future to target colonization of “superbugs” without the need for novel antibiotics.

Marchisio et al. studied adherence and found colonization of the probiotic to be a prerequisite for clinical efficacy. Martensson et al. also studied colonization and found no evidence of colonization of the Honey bee microbiome 1 month after cessation of treatment, which could potentially explain the lack of effect (Mårtensson et al., 2017). This finding also suggests that strains originating from the human airway have a better chance of adherence and clinical efficacy than ever so *in vitro* effective strains derived from other species.

## Thoughts on future interventional studies

What features of a probiotic strain confers positive effects? Is it possible to predict, engineer and enhance these properties in the future? For now, we are not able to answer these questions and the potential role of topical or systemic probiotics in the treatment of CRS is still in its early days. Such treatments could potentially reduce the use of antibiotics and serve as a new treatment alternative, that is both inexpensive and has an excellent safety profile. They could help to reduce antibiotic resistance in society as well as providing an alternative treatment in patients with multi-resistant bacterial colonization. Unfortunately, the complexity and dynamics of the microbiome, in combination with the interaction between the microbiome and the host immune system makes for an incredibly complex situation with endless scenarios possible. At best our assumption on what constitute an effective probiotic treatment is merely an educated guess derived from bacterial interference studies *in vitro*. However, with the very good safety profile of probiotic bacterial strains there is scope to perform trial and error clinical trials and forego extensive laboratory testing. One such successful empirical trial is the fecal transplant to treat Clostridium difficile infection, published in *New England Journal of Medicine*, interim analysis revealed the efficacy of the fecal transplant with resolution in 15 of 16 patients in the transplant group and 4 of 14 in the vancomycin group. No surprise that the study was stopped at the interim analysis (van Nood et al., 2013). To my knowledge successful transplant of nasal mucosal secretions has yet to be published. But it is an intriguing avenue to explore.

## Probiotic trial design in CRS

There are several choices to make when designing a probiotic study. I will discuss some of the aspects in the section below.

### Living or dead bacteria?

For antibacterial interference, it is likely that living bacteria with the capacity to produce antibacterial compounds would be more beneficial. Dead bacteria where the mechanism of action is limited to bacterial surface structures interacting with host immune receptors in a beneficial way, will not incur the risk of developing resistance or virulence genes. However, living probiotics have so far shown to be very safe and from that perspective limit use to dead bacteria is not necessary.

### Single strain or multiple strains?

Although there is an example of a successful single strain intervention (Marchisio et al., 2016), the general impression is that multiple strains have the potential to provide synergistic effects. However, it requires further testing in the laboratory to make sure that the strains do not counteract each other while in the delivery device. Like mixing the wrong fish in the fish tank.

### Prevention or treatment?

In adult CRS, we are of course focusing on treatment, but one could speculate that for example postoperatively provide patients with probiotic nasal washes to prevent a shift toward a gram-negative microbiome or to down-regulate an excess growth of *S. Aureus*. Attenuation of eosinophilic inflammation is another possibility and will be discussed below.

### Probiotics as single treatment modality or adjuvant to antibiotics?

To facilitate the effect of probiotic supplement, a reduction of the load of bacterial pathogens by antibiotic treatment seems like the preferred route. One could start the probiotic supplement simultaneously with antibiotics and then continue after cessation of antibiotic treatment to prevent, with the probiotic, any potential for pathogen regrowth.

### Local treatment or systemic treatment?

Using probiotics as living antibiotics, in other words bacterial interference, in form of a topical wash or spray, has in my personal opinion, the potential to be more effective rather than acting by proxy through the immune system. Dual action by both bacterial interference and immune stimulation is likely to be of benefit and it is not unlikely that any nasal probiotic spray or wash will to some extent end up in the gut with the potential to interact with the extensive lymphoid tissue present in the gastrointestinal tract conferring an added effect on the immune system enabling a positive effect on the gut-lung axis.

### Other aspects of trial design?

To develop our understanding of the mechanisms involved, any probiotic trial design should include a detailed microbiology follow up where robust methods to monitor the strains used. Furthermore, a comprehensive collection and analysis of inflammatory parameters is paramount. Most important however, is a well-defined patient cohort, where one must forego the present nomenclature and more precisely define the pathophysiological mechanisms responsible for the CRS phenotype and rather investigate small homogenous cohorts, as opposed to large heterogeneous CRS populations, where results have a tendency to be diluted, sprawling and inconclusive.

## What outcomes to expect

### Reducing flare ups and exacerbations and bacterial complications to the common cold

There is now evidence that even in a person with no symptoms of sinusitis, pathogens are present on the mucosa, although in low abundance. In an experiment where healthy subjects where infected with rhinoviruses, a 5-fold increase was seen in the abundance of Haemophilus Influenza during the following days (Allen et al., 2014). This suggests that a bacterial complication to a viral rhinitis may not require any external source of Haemophilus, but rather created by an imbalance of the microbiome, prompted by rhinovirus exposure. It is not unlikely that a topical probiotic spray with the capacity for bacterial interference against the common respiratory bacterial pathogens will be able to reduce bacterial complications of the common cold or reduce the number of bacterial flare ups in CRS.

### Improving symptoms in chronically infected CRS patients by decolonization of pathogens

Patients with previous multiple sinus surgery procedures tend to develop, or may already prior to surgery, have a high abundance of *S. Aureus* and / or a gram-negative microbiome. A probiotic nasal wash with commensals capable of interfering with for example *Pseudomonas* has the potential to reduce the abundance of the pathogens and subsequently improve patient symptoms and reduce remodeling of the upper airway mucosa. Of concern, is the presence of intra-cellular *S. Aureus*, which may or may not be the driver of symptoms, but would probably not be accessible to interaction by a probiotic topical spray (Tan et al., 2014).

### Reduce eosinophilic inflammation in the airway

Perhaps the most intriguing concept which, if successful, could have a big impact on asthma as well. This approach may include systemic probiotics influencing the gut-lung axis (Marsland et al., 2015). The gut-lung axis is poorly understood, but suggests that there is a considerable cross talk between the gut and the airway through the immune system. A strong correlation has been made between low microbial diversity in the gut during early infancy and an asthma in childhood (Bisgaard et al., 2011). It is believed that systemic probiotic supplement stimulates dendritic cell maturation and induce a Th1 response through interleukin-12 and interferon-gamma. A recent review summarized the findings of preventing allergic rhinitis by systemic probiotics and showed that out of 23 studies comprising of 1991 patients, 17 studies showed benefit whereas 6 showed no benefit (Zajac et al., 2015). A more recent review revealed similar result but added that all 5 studies with *Lactobacillus paracasei* demonstrated clinically significant improvement suggesting that this is an important probiotic (Guvenc et al., 2016). The results regarding eczema is also encouraging, however the result of systemic probiotic supplement in asthma has been less convincing. A study in patients with eosinophilic CRS treated with systemic probiotics aimed to induce a shift in the immune system from Th2 to Th1 would be of particular interest.

### Can we prevent the development of eosinophilic inflammation?

The ultimate goal, to manipulate the microbiome early in life, according to the hygiene hypothesis, by adding probiotic strains that stimulates development of the Th1 pathway, preventing the development of excess eosinophilic inflammation in the airway. One such pathway that looks promising may be the use of hookworms (Navarro et al., 2016). Whatever type of probiotics, if successful, it will have a large impact on health, and healthcare costs, especially in the developed world, which have seen a surge in inflammatory disease over the last half a century.

## Conclusion

Proof of concept exists for topical probiotic treatment of recurrent infections in the upper airway. However, there are also studies without clinical effect. The complexity of the microbiome and its interaction with the host immune system makes this a very challenging research area. A strong collaboration between ENT surgeons, respiratory physicians, microbiologists and researchers in inflammation and immunity is paramount. It is my personal view that we need to engage in empirical interventional studies, rather than trying to unravel mechanisms in the laboratory or spend too much resources on descriptive work. If successful, probiotics could provide a highly valued, inexpensive and safe treatment of airway disease, and is likely to have the added benefit of reducing antibiotic prescriptions and thus contribute to tackling the rising incidence of antibiotic resistance.

## Author contributions

All authors listed have made a substantial, direct and intellectual contribution to the work, and approved it for publication.

### Conflict of interest statement

The author declares that the research was conducted in the absence of any commercial or financial relationships that could be construed as a potential conflict of interest.
